# TP63 transcriptionally regulates SLC7A5 to suppress ferroptosis in head and neck squamous cell carcinoma

**DOI:** 10.3389/fimmu.2024.1445472

**Published:** 2024-08-21

**Authors:** Zilong Chen, Haoxi Cai, Weiwei Ye, Junming Wu, Jing Liu, Yun Xie, Shiqiang Feng, Yuanpei Jin, Yunxia Lv, Hui Ye, Chengfu Cai, Gengming Cai

**Affiliations:** ^1^ Department of Otolaryngology-Head and Neck Surgery, Xiamen Medical College Affiliated Haicang Hospital, The Sixth Hospital of Xiamen City, The Haicang Hospital of Xiamen, Xiamen, China; ^2^ Department of Oncology, Quanzhou First Hospital Affiliated to Fujian Medical University, Quanzhou, China; ^3^ Xiangya Stomatological Hospital, Xiangya School of Stomatology, Central South University, Changsha, China; ^4^ Experimental Center of BIOQGene, YuanDong International Academy of Life Sciences, Hong Kong, Hong Kong SAR, China; ^5^ Systems Biology Research Center, Biology Institute, Guangxi Academy of Sciences, Nanning, Guangxi, China; ^6^ Department of Thyroid and Head and Neck Surgery, Nanchang University Second Affiliated Hospital, Nanchang, China; ^7^ Department of Clinical Medical, Fujian Medical University, Fuzhou, China

**Keywords:** head and neck squamous cell carcinoma, ferroptosis, single-cell transcriptomics, immunotherapy, tumor microenvironment

## Abstract

**Background:**

Most head and neck squamous cell carcinoma (HNSCC) patients are diagnosed at an advanced local stage. While immunotherapy has improved survival rates, only a minority of patients respond durably to targeted immunotherapies, posing substantial clinical challenges. We investigated the heterogeneity of the tumor microenvironment in HNSCC cohorts before and after immunotherapy by analyzing single-cell RNA sequencing (scRNA-seq) data and bulk RNA sequencing datasets retrieved from public databases.

**Methods:**

We constructed a single-cell transcriptome landscape of HNSCC patients before and after immunotherapy and analyzed the cellular composition, developmental trajectories, gene regulatory networks, and communication patterns of different cell type subpopulations. Additionally, we assessed the expression levels of relevant indicators in HNSCC cells via western blot, ELISA, and fluorescent probe techniques.

**Results:**

At the single-cell level, we identified a subpopulation of TP63^+^ SLC7A5^+^ HNSCC that exhibited a ferroptosis-resistant phenotype. This subpopulation suppresses ferroptosis in malignant cells through the transcriptional upregulation of SLC7A5 mediated by high TP63 expression, thereby promoting tumor growth and resistance to immunotherapy. The experimental results demonstrated that the overexpression of TP63 upregulated the expression of SLC7A5 and suppressed the concentrations of Fe^2+^ and ROS in HNSCC cells. By integrating bulk transcriptome data, we developed a clinical scoring model based on TP63 and SLC7A5, which are closely associated with tumor stage, revealing the significant prognostic efficacy of the TP63^+^ SLC7A5^+^ HNSCC-mediated ferroptosis mechanism in HNSCC patients.

**Conclusion:**

Our research elucidates the TME in HNSCC before and after immunotherapy, revealing a novel mechanism by which TP63^+^ SLC7A5^+^ HNSCC inhibits ferroptosis and enhances tumor resistance via TP63-induced SLC7A5 upregulation. These insights lay the foundation for the development of more effective treatments for HNSCC.

## Introduction

Head and neck squamous cell carcinoma (HNSCC), which develops from the mucosal epithelium of the oral cavity, pharynx, and larynx, ranks as the sixth most common malignancy worldwide (1, 2). Studies indicate that approximately 60% of HNSCC patients are diagnosed at a locally advanced stage, and despite receiving treatments, up to 50% of patients experience local recurrence or distant metastasis (3). Immunotherapy has been shown to induce changes in the tumor microenvironment, increasing sensitivity to subsequent treatments (4); however, fewer than 20% of HNSCC patients exhibit a durable response to targeted immunotherapies (2). Currently, significant advancements have been achieved in multimodal treatment strategies for HNSCC, including ablative surgery, chemotherapy-radiotherapy, and immunotherapy (5). However, the efficacy of these treatments is limited by various factors, including tumor heterogeneity and the complexity of the immune microenvironment (2, 5). Therefore, understanding the evolutionary mechanisms of tumor cells and enhancing patients’ antitumor responses are essential for developing new immunotherapeutic approaches that target HNSCC cells.

Ferroptosis is a nonapoptotic form of programmed cell death, with tumor cell ferroptosis shown to inhibit tumor progression (6). Owing to the unsatisfactory clinical prognosis and treatment outcomes of HNSCC patients and given the susceptibility of ferroptosis to modulation by various combination therapies, the activation of ferroptosis has garnered significant attention as a promising therapeutic strategy in recent years (6). Previous research has revealed a spatial correlation between ferroptosis and the expression of transcriptional markers of inflammation and immune activation at the invasion fronts of HNSCC (7). Furthermore, ferroptosis enhances antitumor immune responses by inducing immunogenic exposure in HNSCC (8). However, our understanding of the molecular mechanisms underlying ferroptosis in HNSCC, particularly at the single-cell level, is still limited. Thus, the use of single-cell RNA sequencing (scRNA-seq) to analyze gene expression in individual cells (5) is crucial for revealing the heterogeneity of ferroptosis-related cellular subpopulations in HNSCC and identifying potential therapeutic targets for ferroptosis.

In this study, by combining scRNA-seq with bulk transcriptome data, we analyzed the dynamic changes in tumor heterogeneity and the TME before and after immunotherapy in patients with HNSCC, providing a comprehensive single-cell transcriptomic landscape for human HNSCC. Through the analysis of transcriptional features, our study identified a malignant cell subpopulation that exhibited a ferroptosis-resistant phenotype, and experimental validation confirmed this finding. In summary, we have improved our understanding of the heterogeneity and complexity of the TME in HNSCC, potentially advancing its application in the personalized treatment of HNSCC.

## Methods

### Material sources

The scRNA-seq data related to HNSCC were obtained from the Gene Expression Omnibus (GEO) (https://www.ncbi.nlm.nih.gov/geo/) with the accession number GSE195832 (9). The data are based on the GPL24676 platform, which includes four patient tumors before and after treatment from a neoadjuvant trial of advanced-stage HNSCC patients who were treated with the aPD-1 therapy nivolumab. Moreover, the transcriptome data for HNSCC utilized in this study, the Cancer Genome Atlas-Head and Neck Squamous Cell Carcinoma (TCGA-HNSC), were sourced from the TCGA database (https://portal.gdc.cancer.gov/projects/TCGA-HNSC), comprising 502 HNSCC samples, 44 adjacent normal controls, and associated clinical information.

### ScRNA-seq data and bulk RNA-seq data preprocessing

Using the Seurat package (10), this study applied filters to remove cells whose gene expression was in the top and bottom 1% and those whose mitochondrial gene expression was greater than 10%. Single-cell unique molecular identifier (UMI) expression data were normalized via regularized negative binomial regression. The Seurat package was subsequently used to refine and integrate HNSCC scRNA-seq data, facilitating cell dimension reduction and clustering. Clustering results were visualized through the uniform manifold approximation and projection (UMAP) algorithm (11).

For TCGA-HNSC data normalization and preprocessing, we used the normalizeBetweenArrays function in the limma software package (12).

### Identification of cellular clusters

This study utilized the FindAllMarkers function from the Seurat package to identify genes that were differentially expressed between specific cell clusters and other clusters (P value < 0.05). The cell types were manually annotated on the basis of cell marker databases, laboratory expertise, and previous research publications (13–15). We subsequently performed cluster analysis on various types of cells, manually annotating cell subpopulations within each cell type on the basis of genes that were significantly representative and functional, indicating specificity.

### Estimating the immune response of the system

This study utilized the easier package (16) to predict the level of immune response in HNSCC samples. On the basis of the results of the Easier score, samples were classified according to the median Easier score: those exceeding the median value were designated the immunotherapy responsive (IR) group, whereas those below the median were assigned to the immunotherapy non-responsive (INR) group. Detailed grouping information for HNSCC samples is provided in **Supplementary Table S1**.

### Enrichment analysis and gene set enrichment analysis

To uncover significant biological patterns, enrichment analysis targets closely related biological phenomena, pinpointing common or unique biological features across cell subpopulations. This research utilized the clusterProfiler R package (17) to perform enrichment analyses on biological processes (BP) and the Kyoto Encyclopedia of Genes and Genomes (KEGG), adopting a P value threshold of < 0.05 for statistical significance.

Gene set enrichment analysis (GSEA) (18) was employed to assess the distribution of genes across an expression profile, categorized by phenotypic relevance. Using reference genomes from MsigDB V7.4, specifically c5.bp.v7.0.entrez.gmt and c2.cp.kegg.v7.0.symbols.gmt (19), we conducted an in-depth analysis of gene expression and phenotypic profiles within various cellular subpopulations. Additionally, we employed the AUCell function (https://bioconductor.org/packages/release/bioc/html/AUCell.html) to perform cell scoring on specified gene sets, thereby identifying the expression profiles of gene sets associated with active pathways at the cellular subpopulation level.

### Inference of differentiation status in cell subpopulations

Single-cell transcriptional diversity is a powerful indicator of developmental potential within a cell population. To gain further insight into the cellular dynamics and potential progression patterns of malignant cells, this study employed the R package CytoTRACE (20) to infer the differentiation state of HNSCC cell subpopulations.

### Trajectory analysis

In response to external stimuli or throughout developmental stages, cells exhibit a range of functional alterations, manifested in distinct expression profiles. By categorizing cells according to the trajectories of their gene expression changes, it becomes possible to delineate their developmental courses. In this study, the R package Monocle2 (21, 22) was utilized to construct developmental trajectories within subpopulations of different cell types, with visualization facilitated by the UMAP algorithm. This approach offers detailed insight into the dynamics of cellular evolution and differentiation.

### Gene regulatory network analysis

Transcription factors (TFs) shape gene expression, impacting an organism’s physiological functions by modulating specific gene expression levels and patterns. To identify the specific TFs regulating each cell subpopulation, an analysis and reconstruction of the TF-centered gene regulatory network (GRN) (23, 24) was conducted via the pySCENIC python module tool. Additionally, transcription factor binding profiles were obtained from the JASPAR database (available at https://jaspar.genereg.net) to establish patterns of transcription factors regulating each coexpressed gene module.

### Gene set variation analysis

Gene set variation analysis (GSVA) transforms the gene expression matrix across various samples into a matrix of gene set expression across samples, enabling the assessment of disparities in distinct gene sets across samples. In this study, the top 15 genes with significant and specific expression in the different cell subpopulations were chosen as the gene set. The single-sample gene set enrichment analysis (ssGSEA) method from the GSVA package (25) was employed to compute the scores of different subgroups in the TCGA-HNSC cohort, and the connections between cell subpopulation abundance and clinical prognosis characteristics were subsequently analyzed. On the basis of the algorithm-derived optimal cutoff, the samples were stratified into high-score and low-score groups, and the R packages survival (https://cran.r-project.org/web/packages/survival/index.html) and survminer (https://rdocumentation.org/packages/survminer/versions/0.4.9) were subsequently utilized to determine the overall survival (OS) rate and recurrence-free survival (RFS) rate. The R package pROC was used to analyze the receiver operating characteristic (ROC) curve (26).

### Cox univariate and multivariate analyses

To determine whether our model can be used as an independent predictor for HNSCC patients, we defined genes with downregulated ferroptosis signaling pathways in TP63^+^ SLC7A5^+^ HNSCC patients as TP63^+^ SLC7A5^+^ HNSCC-related ferroptosis genes. Together with TP63 and SLC7A5, these genes were amalgamated to formulate the scoring genes. We subsequently conducted both univariate and multivariate Cox regression analyses (27) on these scoring genes. We constructed a clinical scoring model based on the mechanism score through Cox regression analyses (27), integrating the clinical indicators of HNSCC patients. We subsequently used the “rms” R package (28) to create column line plots for predicting one-, three-, and five-year OS in clinical patients to evaluate the potential of the model for predicting the prognosis of HNSCC patients. Finally, calibration curves were employed to assess the accuracy of the model by comparing the expected and observed survival rates.

### Cell communication analysis

The iTALK package (https://doi.org/10.1101/507871) identifies cell-specific highly or differentially expressed ligand−receptor genes, recognizing potential communication pathways that may play crucial roles in tumor progression. In this study, we utilized the iTALK package to uncover noteworthy ligand−receptor interactions among subpopulation cells, thus offering an intricate view of intercellular communication dynamics during developmental processes.

### HNSCC cell line

The human HNSCC cell line SCC25 was acquired from Pricella (CM-0569) and propagated in Dulbecco’s modified Eagle’s medium/nutrient mixture F-12 (DMEM/F12, Gibco, 25200072), supplemented with 10% fetal bovine serum (FBS, Solarbio, P1020), 400 ng/mL hydrocortisone, and 1% penicillin/streptomycin solution. The cells were cultured at 37°C in a humidified incubator under an atmosphere containing 5% carbon dioxide.

### Synthesis of the TP63-specific siRNA sequence and siRNA transfection

The small interfering RNA (siRNA) sequence targeting human TP63 was synthesized by Sangon Biotech (Shanghai, China), with the following sequence information:

Sense: 5’-UGGAUUUGUACCAUUCUUCUGTT-3’.

Antisense: 5’-GAAGAAUGGUACAAAUCCAAGTT-3’.

The HNSCC cell line SCC25 was seeded into a six-well plate and allowed to grow until 60% to 70% confluence was achieved. The old medium was then discarded, and the cells were washed twice with phosphate-buffered saline (PBS). Each well was subsequently supplemented with 2 mL of DMEM/F-12. Two sterile centrifuge tubes were prepared, each containing 250 μL of DMEM/F-12; 200 pmol of siRNA was added to one tube, and 10 μL of Lipo6000™ transfection reagent (Beyotime, C0526) was added to the other. Both tubes were allowed to stand at room temperature for 5 minutes. The siRNA mixture was then gently mixed into a tube containing Lipo6000™ transfection reagent, and the mixture was incubated at room temperature for an additional 5 minutes. After transfection, the cells were incubated in the original medium for 4 hours before the medium was replaced with fresh DMEM/F-12 containing 10% FBS, 400 ng/mL hydrocortisone, and 1% penicillin/streptomycin.

### Construction of the TP63 overexpression plasmid and lipofection

The TP63 overexpression plasmid was constructed by Sangon Biotech, utilizing the pcDNA3.1 vector with NheI and BamHI as the restriction sites.

The HNSCC cell line SCC25 was seeded into a six-well plate and grown until 60% to 70% confluence was achieved. The old medium was subsequently discarded, and the cells were subsequently rinsed twice with PBS. Each well was replenished with 2 mL of DMEM/F-12. Two sterile centrifuge tubes were prepared, each containing 250 μL of DMEM/F-12; one tube was supplemented with 5 μg of the pcDNA3.1-TP63 overexpression plasmid, and the other was supplemented with 10 μL of Lipo6000™ transfection reagent. Both tubes were left at room temperature for 5 minutes. The solution containing the pcDNA3.1-TP63 plasmid was then gently mixed into the tube with Lipo6000™ transfection reagent, and the mixture was incubated at room temperature for an additional 5 minutes. After transfection, the cells were incubated in the original medium for 4 hours before the medium was replaced with fresh DMEM/F-12 containing 10% FBS, 400 ng/mL hydrocortisone, and 1% penicillin/streptomycin.

### Western blot

To assess the protein expression levels of P63 and SLC7A5, Western blot assays were performed with the following primary antibodies: anti-human p63 (ab32353, Abcam; 1:10000) and anti-human SLC7A5/LAT1 (ab305251, Abcam; 1:1000). Initially, gels were cast with an acrylamide solution and polymerized via TEMED. The samples were combined with SDS loading buffer, denatured and electrophoresed, followed by transfer onto PVDF membranes. These membranes were subsequently blocked and incubated with primary antibodies overnight and then with HRP-conjugated secondary antibodies. Detection was facilitated by enhanced chemiluminescence, with imaging conducted on a Bio-Rad CHEMIDOC XRS^+^ imaging system. For sequential antigen detection, the membranes underwent a stripping process before reprobing with new antibodies, ensuring thorough analysis of protein expression.

### Measurement of iron ion concentrations via ELISA

The required strip was retrieved from an aluminum foil bag after equilibration at room temperature for 20 minutes, and any unused strips were stored at 4°C. Standard and sample wells were prepared by adding 50 μL of standards at concentrations of 0, 15, 30, 60, 120, and 240 ng/mL to standard wells. A total of 10 μL of sample and 40 μL of diluent were added to the sample wells, and the blank wells were left empty. One hundred microliters of HRP-conjugated detection antibody was added to each nonblank well, and the samples were sealed with a cover film and incubated at 37°C for 60 minutes. The reaction mixture was removed, the mixture was patted dry, washing buffer was added, and the mixture was allowed to sit for 1 min. The mixture was discarded, and the mixture was patted dry. This process was repeated 5 times. Then, 50 μL of substrates A and B from the iron ion assay kit were added to each well and incubated in the dark at 37 μL for 15 minutes. After 50 μL of stop solution was added, the OD was measured at 450 nm.

### Reactive oxygen species detection

The oxidation-sensitive fluorescent probe (DCFH-DA) was diluted in serum-free culture medium at a ratio of 1:1000, and the collected cells were suspended in diluted DCFH-DA solution and incubated at 37°C in the dark for 20 minutes. The cells were washed three times with serum-free culture medium to thoroughly remove any uninternalized DCFH-DA. The intracellular ROS levels were determined via flow cytometry, with the fluorescence intensity values reflecting the ROS levels.

### Data statistics and analysis

All the bioinformatics analyses and research in this study were conducted on a bioinformatics cloud platform (http://www.bioinforcloud.com). A P value < 0.05 was considered statistically significant.

## Results

### Single-cell transcriptional landscape of HNSCC patients before and after immunotherapy

**Figure 1:**
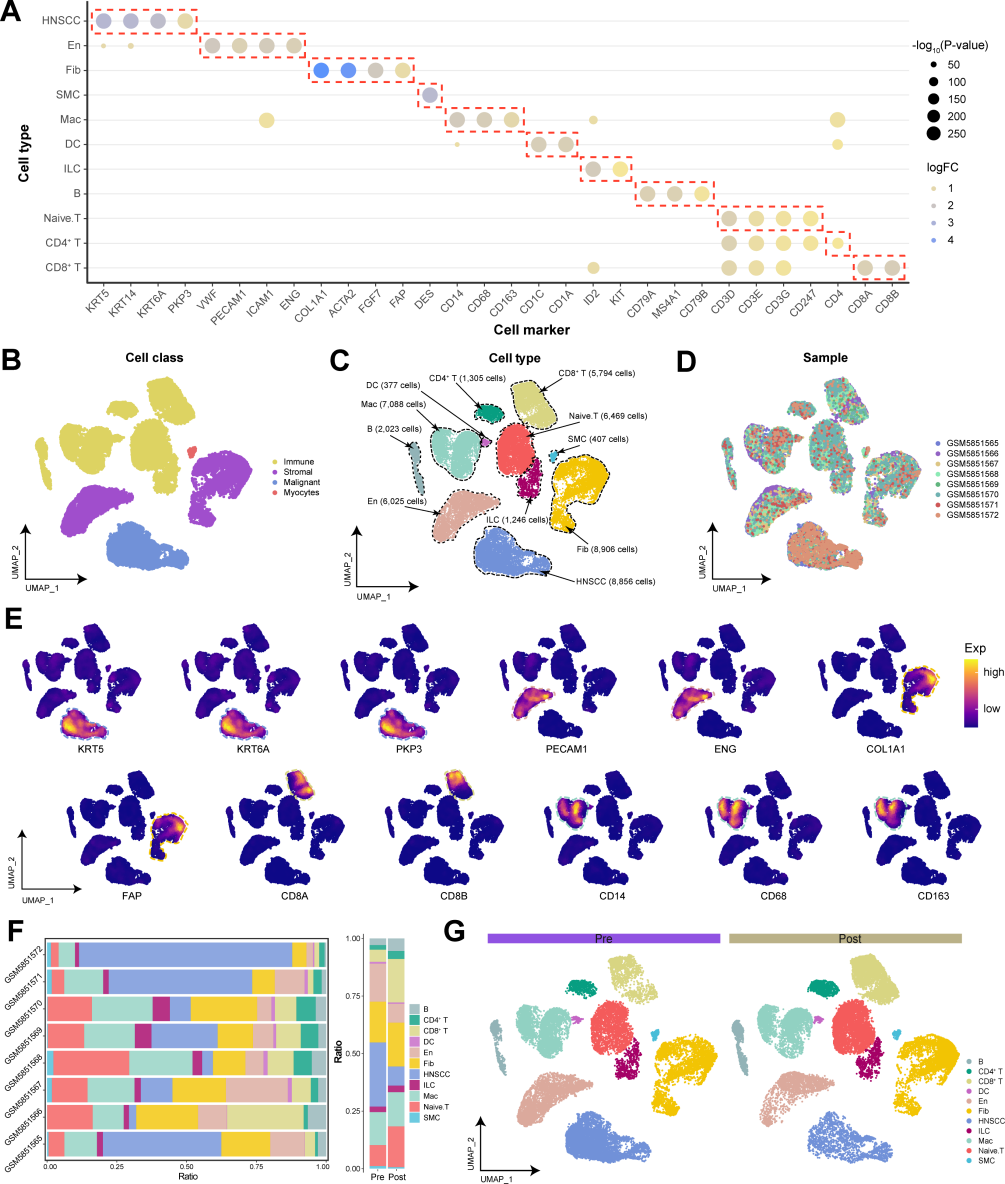
The global single-cell transcriptional landscape of HNSCC patients before and after immunotherapy. **(A)** Specific cell biomarker genes for cell cluster annotation and merging. **(B)** Single-cell transcriptional landscape of major cell classes in HNSCC. **(C)** Single-cell transcriptional landscape of major cell types in HNSCC. **(D)** Single-cell transcriptional landscape of different HNSCC samples. **(E)** Expression of specific biomarker genes in the single-cell transcriptional landscape. **(F)** Dynamic changes in cellular components. (Left) Cellular composition of different HNSCC samples. (Right) Cellular composition of HNSCC patients before and after immunotherapy. (Pre: Pre-treatment, Post: Post-treatment) **(G)** Single-cell transcriptional landscape of different groups of HNSCC patients.

This study used bioinformatics analysis of a total of 56,582 cells from 4 pre-immunotherapy and 4 post-immunotherapy HNSCC samples from the GEO database. Following data preprocessing, 48,457 high-quality single-cell transcriptomes were retained, and 32 cellular clusters were identified through dimensionality reduction and clustering (**Supplementary Table S2**). On the basis of classical cell markers identified in prior studies (13–15), we manually annotated and merged these cell clusters on the basis of the specific cell markers expressed within each cluster and presented the primary markers within each corresponding cell type (**Supplementary Table S3**): HNSCC cells (KRT5, KRT6A, and PKP3), En (endothelial cells: VWF and PECAM1), Fib (fibroblasts: COL1A1 and ACTA2), SMC (smooth muscle cells: DES), Mac (macrophages: CD14 and CD163), DC (dendritic cells: CD1A and CD1C), ILC (innate lymphoid cells: ID2 and KIT), B cells (MS4A1, CD79A and CD79B), Naive. T cells (CD3D, CD3E, CD3G, and CD247), CD4^+^ T cells (CD4), and CD8^+^ T cells (CD8A and CD8B) (**Figure 1A**). We ultimately annotated 4 major cell classes or 11 major cell types and depicted the distribution of different samples across various cell types (**Figures 1B-D**). The expression of specific biomarker genes in the single-cell transcriptional landscape further validated the accuracy of our annotations (**Figure 1E**).

Compared with patients in the pre-immunotherapy state, patients in the post-immunotherapy state presented a significant reduction in malignant and endothelial cells, with a slight increase in Fib abundance (**Figures 1F, G**). This may indicate that immunotherapy has remodeled the TME, diminished angiogenesis, and consequently restricted tumor growth and dissemination. Additionally, the ratio of T cells to B cells significantly increased after treatment, suggesting that immunotherapy further activated the patient’s immune cells, enhancing the immune response against the tumor.

### The TP63 and SLC7A5 double-positive HNSCC cells significantly inhibited the ferroptotic pathway

**Figure 2:**
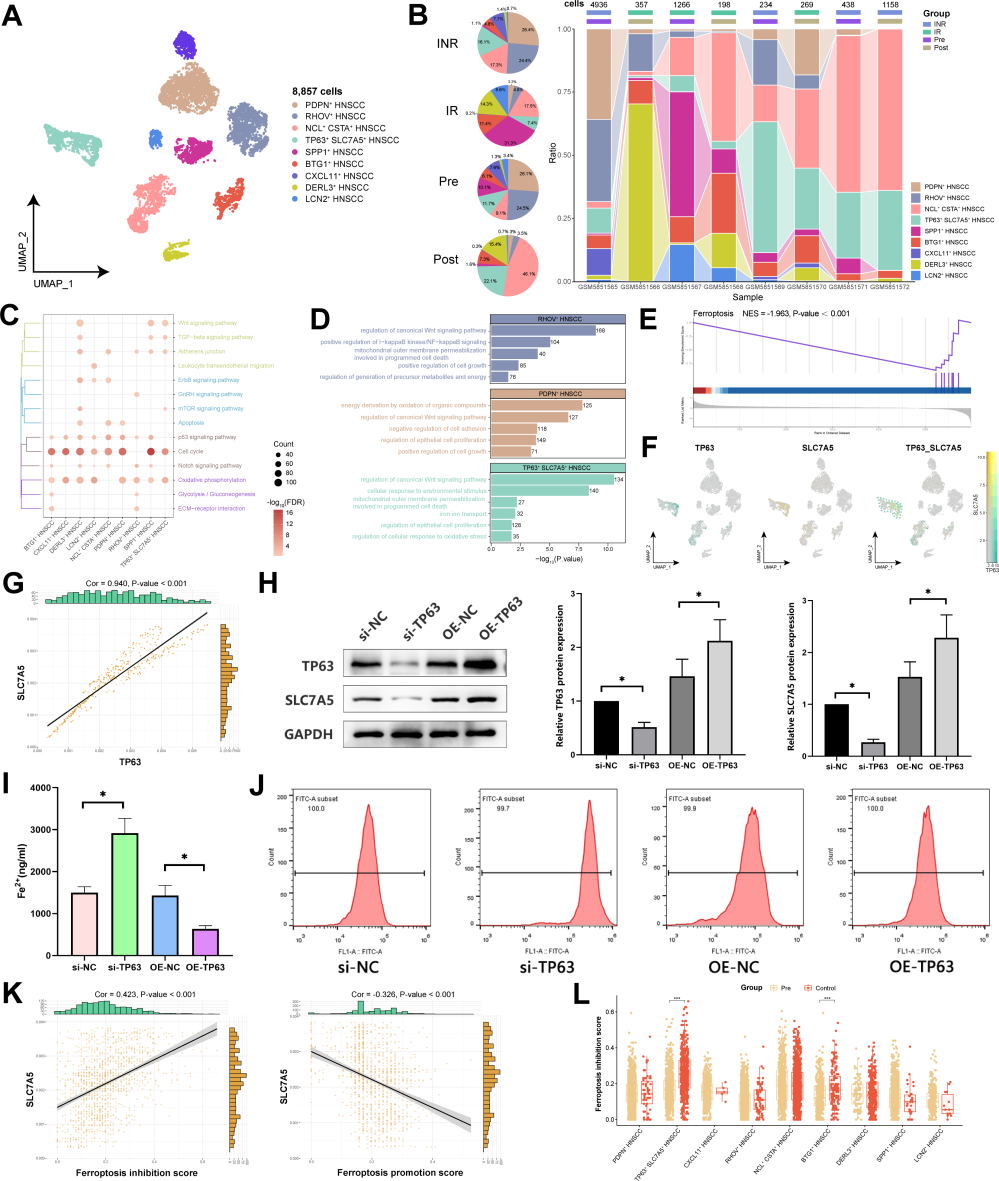
Single-cell transcriptional landscape of HNSCC cells before and after immunotherapy. **(A)** Single-cell transcriptional landscape of HNSCC cell subpopulations. **(B)** Dynamic changes in HNSCC cell subpopulations. (Left) HNSCC cell subpopulations composition of different groups. (Right) HNSCC cell subpopulations composition of different groups. (IN: Immune response, INR: Immune non-response, Pre: Pre-treatment, Post: Post-treatment) **(C)** The signaling pathways associated with HNSCC cell subpopulations. **(D)** The biological processes associated with HNSCC cell subpopulations. **(E)** The TP63^+^ SLC7A5^+^ HNSCC subpopulation significantly inhibited Ferroptosis. **(F)** TP63 and SLC7A5 coexpression in TP63^+^ SLC7A5^+^ HNSCC subpopulation. **(G)** Correlation between TP63 and SLC7A5. **(H)** Western blotting for the protein levels of TP63 and SLC7A5 in each group of HNSCC cells. **(I)** Detection of the Fe^2+^ ion concentration in each group of HNSCC cells. **(J)** Detection of the ROS concentration in each group of HNSCC cells. **(K)** Correlation between SLC7A5 and genes associated with ferroptosis. **(L)** Ferroptosis inhibition scores of HNSCC subpopulations. *, P value < 0.05.

To investigate the functional heterogeneity among different HNSCC subpopulations within the TME, we identified nine HNSCC subpopulations through cluster analysis and annotated each subpopulation on the basis of functionally specific genes that were highly expressed (**Figure 2A**; **Supplementary Figure S1A**). Notably, we identified a malignant cell subpopulation expressing high levels of the key oncogenic driver gene TP63 in squamous cell carcinoma, known as TP63^+^ SLC7A5^+^ HNSCC. Comparing the differences in patients with different states, we found that the subpopulation of TP63^+^ SLC7A5^+^ HNSCC subpopulation increased in abundance following immunotherapy compared with pre-immunotherapy levels and was more abundant in the immune non-responsive group than in the immune-responsive group (**Figure 2B**; **Supplementary Figure S1B**). These findings suggest that TP63^+^ SLC7A5^+^ HNSCC subpopulation may exhibit resistance to immunotherapy.

Our analysis revealed that various HNSCC subpopulations are distinctly enriched in pathways related to energy metabolism and the cell cycle (**Figure 2C**). Notably, TP63^+^ SLC7A5^+^ HNSCC subpopulation presented pronounced enrichment of functions associated with iron ion transport, mitochondrial activities, and cell proliferation, highlighting their unique biological roles (**Figure 2D**). Further investigations revealed substantial inhibition of the ferroptosis signaling pathway in TP63^+^ SLC7A5^+^ HNSCC subpopulation (**Figure 2E**). By delving deeper into this subpopulation’s related mechanisms, we observed that TP63 and SLC7A5 were both coexpressed and strongly positively correlated (Cor = 0.94) in TP63^+^ SLC7A5^+^ HNSCC (**Figures 2F, G**). This finding was substantiated by Western blot analysis, which revealed that the overexpression of TP63 led to a concurrent increase in SLC7A5 levels in HNSCC cells (**Figure 2H**). Moreover, the overexpression of TP63 in HNSCC cells significantly decreased the concentrations of Fe^2+^ and ROS, whereas the knockdown of TP63 significantly increased these concentrations (**Figures 2I, J**). These findings emphasize the critical role of TP63 in inhibiting ferroptosis in HNSCC cells. By integrating a ferroptosis-related gene set, we noted that SLC7A5 expression strongly correlated with ferroptosis inhibition (Cor = 0.42) and inversely correlated with ferroptosis promotion (Cor = -0.33) (**Figure 2K**). Moreover, the predictive analysis of TP63-protein binding to SLC7A5-DNA revealed significant peak associations (**Supplementary Figure S1C**). Importantly, post-immunotherapy, the ferroptosis score of TP63^+^ SLC7A5^+^ HNSCC subpopulation was markedly different from that of the pre-treatment group (**Figure 2L**; **Supplementary Figure S1D**), suggesting that malignant cells may mediate ferroptosis to resist immunotherapy.

**Figure 3:**
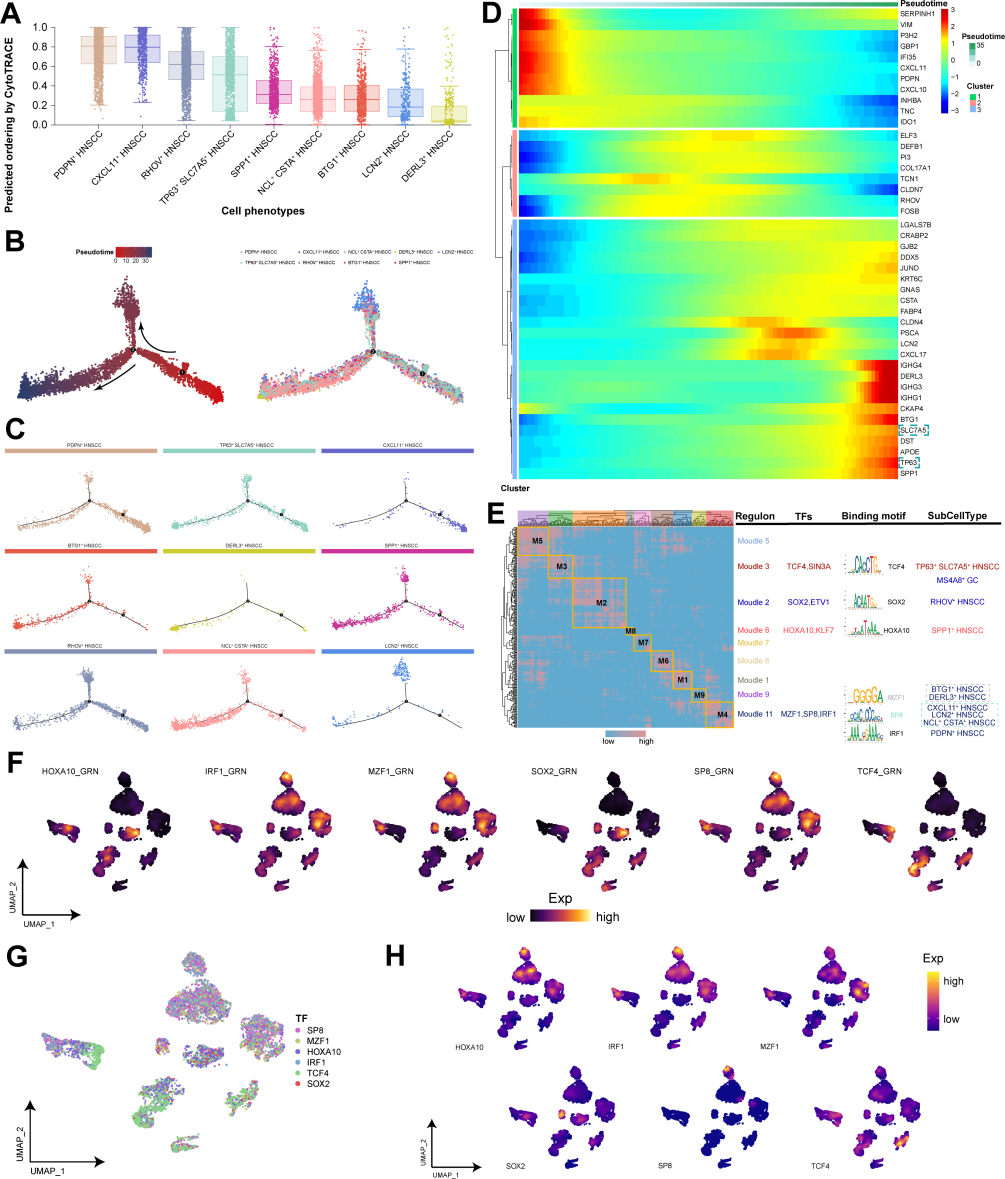
Trajectory analysis and GRN analysis of HNSCC subpopulations. **(A)** Inference of the differentiation status of HNSCC cell subpopulations. **(B)** Trajectory analysis of HNSCC cell subpopulations. (Left) Pseudotime analysis mapping of HNSCC cell subpopulations. (Right) Pseudotime values displaying the differentiation trajectory of HNSCC cell subpopulations. **(C)** Pseudotime analysis mapping the various HNSCC cell subpopulations. **(D)** The dynamics of the top 5 differentially expressed genes in various HNSCC cell subpopulations. **(E)** Motif modules for HNSCC cell subpopulations. **(F)** AUCell scores for the main transcription factors. **(G)** Single-cell transcriptional landscape of the main transcription factors in HNSCC cell subpopulations. **(H)** Expression levels of the main transcription factors.

On the basis of the inferred differentiation of subpopulations within HNSCC, the results indicated that CXCL11^+^ HNSCC and PDPN^+^ HNSCC subpopulations possessed the highest potential for differentiation and may be the developmental origins of HNSCC cells (**Figure 3A**). Through trajectory analysis, our study further revealed the differentiation trajectory of HNSCC subpopulations, with TP63^+^ SLC7A5^+^ HNSCC subpopulation being prevalent throughout the cell differentiation process (**Figures 3B, C**). These findings suggest that this subpopulation possesses high differentiation potential and plasticity, which aligns with our predicted outcomes of differentiation (**Figure 3A**). We subsequently explored the pseudotime differential gene expression among the subpopulations, with TP63 and SLC7A5 expressed in the later stages of the trajectory (**Figure 3D**; **Supplementary Figure S1E**), indicating their significant roles in the maturation and differentiation of malignant cells. GRN analysis illustrated the clustering of regulators within HNSCC cells, identifying TCF4 as a potential key player in the targeted regulation of TP63^+^ SLC7A5^+^ HNSCC subpopulation (**Figures 3E-H**; **Supplementary Figure S1F**). Overall, we discovered a unique self-protection mechanism evolved by malignant cells: TP63 regulates SLC7A5 to inhibit ferroptosis, sustain tumor growth and development, and resist immunotherapy.

### The clinical scoring model for HNSCC based on TP63 and SLC7A5 demonstrated robustness

**Figure 4:**
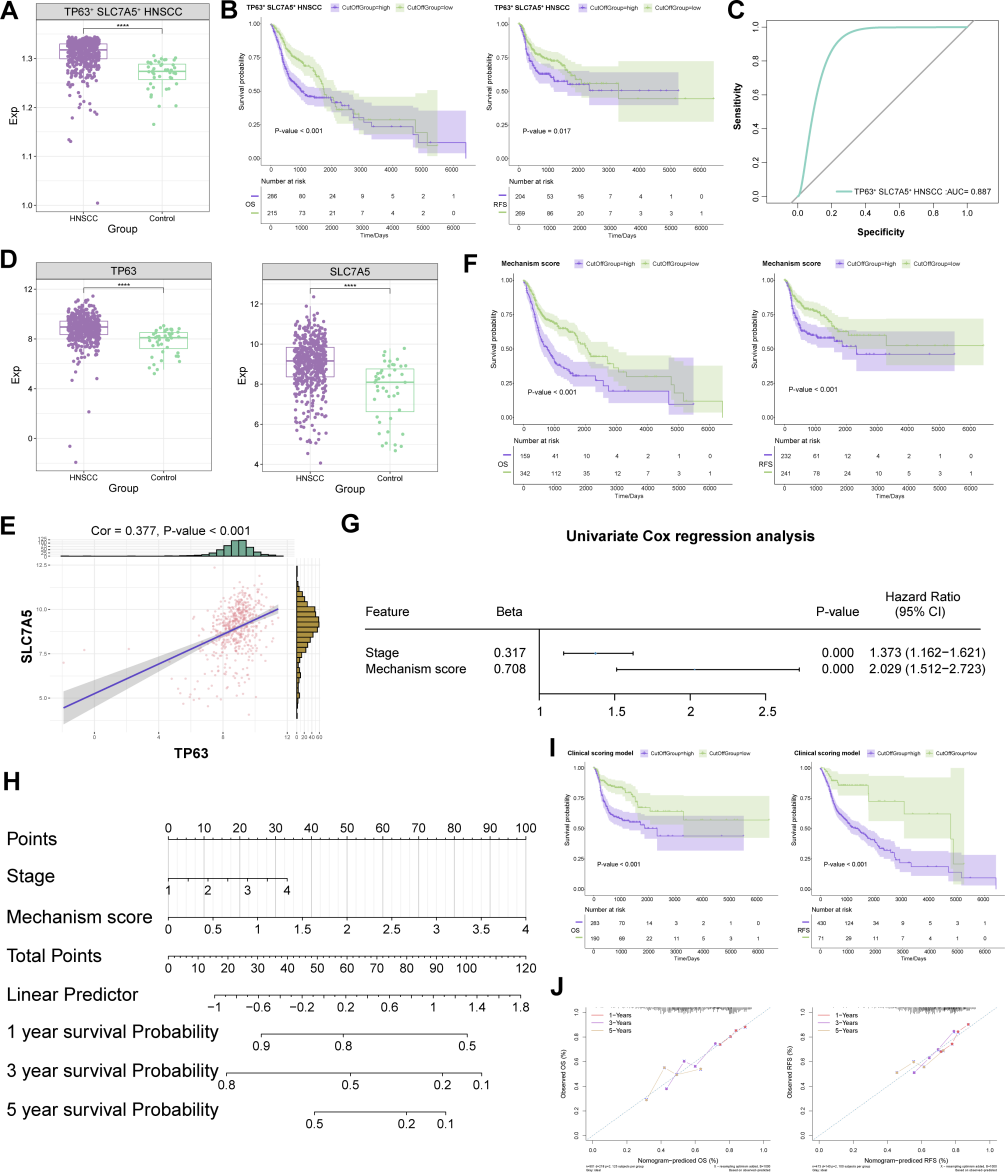
Regulatory mechanisms of the TP63 and SLC7A5 double-positive subpopulation in HNSCC. **(A)** Abundance variations of TP63^+^ SLC7A5^+^ HNSCC in HNSCC patient tissues. **(B)** Prognostic efficacy of TP63^+^ SLC7A5^+^ HNSCC for OS and RFS. **(C)** Diagnostic potential of TP63^+^ SLC7A5^+^ HNSCC for HNSCC patients. **(D)** Expression of TP63 and SLC7A5 in HNSCC patients. **(E)** Correlation between TP63 and SLC7A5 in HNSCC patients. **(F)** Prognostic efficacy of mechanism score for OS and RFS. **(G)** Univariate prognostic efficacy of mechanism score and clinical indicators. **(H)** Prediction of outcomes in HNSCC patients via the clinical scoring model. **(I)** Prognostic efficacy of the clinical scoring model for OS and RFS. **(J)** Accuracy of the clinical scoring model in predicting OS and RFS in the HNSCC clinical patient cohort. ****, P value < 0.0001.

Using bulk transcriptome data from the TCGA database, GSVA revealed that the abundance of TP63^+^ SLC7A5^+^ HNSCC subpopulation significantly increased within HNSCC, contributing to adverse patient outcomes, while exhibiting excellent diagnostic efficacy for identifying HNSCC patients (AUC = 0.887) (**Figures 4A-C**). We subsequently explored the critical roles of TP63 and SLC7A5 in the context of HNSCC patients. Notably, both TP63 and SLC7A5 exhibited elevated expression levels in HNSCC patients compared with controls, demonstrating a notable positive correlation (Cor = 0.38) (**Figures 4D, E**). We subsequently selected the genes TP63 and SLC7A5, as well as genes with downregulated ferroptosis signaling pathways in TP63^+^ SLC7A5^+^ HNSCC subpopulation, as scoring genes (**Supplementary Table S4**). A univariate analysis incorporating scoring genes was conducted to calculate a mechanism score, revealing that a higher score could be indicative of poor prognosis in HNSCC patients (**Figure 4F**). Assessment of the relationships between distinct characteristics and the risk of patient survival revealed significant univariate prognostic efficacy for the mechanism score and tumor stage in HNSCC patients (**Figure 4G**). By integrating the mechanistism score with tumor stage, we developed a clinical scoring model based on TP63 and SLC7A5, employing nomograms for the prediction of one-year, three-year, and five-year survival rates in patients (**Figure 4H**). Survival curve analysis confirmed the superior prognostic predictive ability of the TP63 and SLC7A5 clinical scoring models for HNSCC prognosis (**Figure 4I**). Finally, the prognostic efficacy for HNSCC patients was further substantiated by calibration curves, verifying the accuracy of our predictions (**Figure 4J**).

### NDFA4L2-positive cancer-associated fibroblasts exhibited characteristics of myofibroblasts

**Figure 5:**
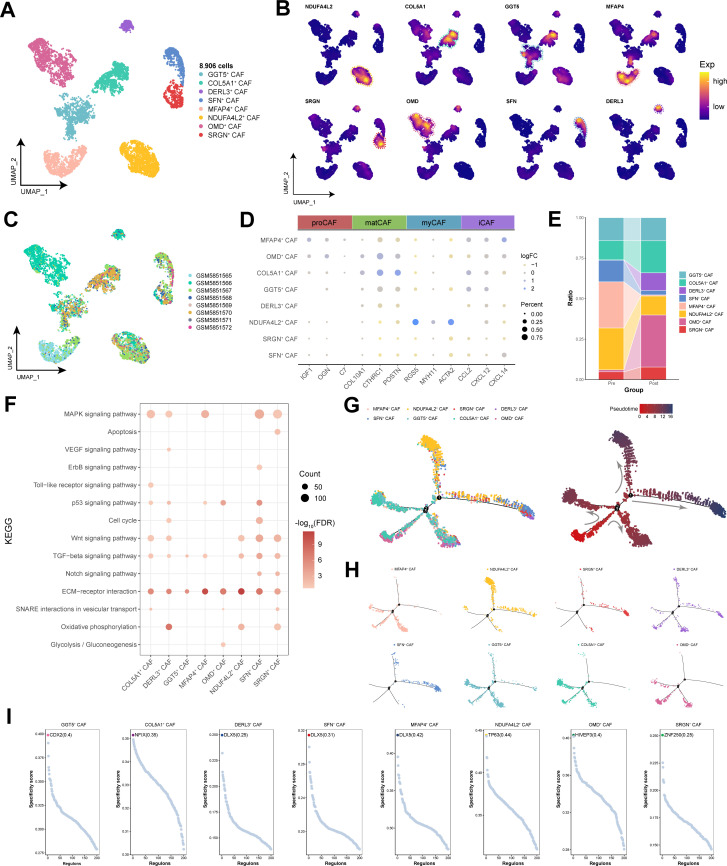
Single-cell transcriptional landscape of CAFs before and after immunotherapy. **(A)** Single-cell transcriptional landscape of CAF subpopulations. **(B)** Expression of specific genes in CAF subpopulations. **(C)** Single-cell transcriptional landscape of CAF subpopulations among different HNSCC samples. **(D)** Marker genes for different CAF subtypes. **(E)** CAF subpopulation compositions of different groups. (Pre: Pre-treatment, Post: Post-treatment) **(F)** The signaling pathways associated with CAF subpopulations. **(G)** Trajectory analysis of CAF subpopulations. (Left) Pseudotime analysis mapping of CAF subpopulations. (Right) Pseudotime values displaying the differentiation trajectory of CAF subpopulations. **(H)** Pseudotime analysis mapping the various CAF subpopulations. **(I)** Specific regulators with the highest transcriptional activity in different CAF subpopulations.

Through clustering analysis of cancer-associated fibroblasts (CAFs), a total of eight distinct cell subpopulations were identified (**Figures 5A-C**). Previous pancancer analyses of CAFs revealed a diverse lineage spectrum, further differing into four subtypes: proCAFs, matCAFs, myCAFs, and iCAFs (29). Within HNSCC, we observed that NDUFA4L2^+^ CAF subpopulation highly expressed the myCAF marker genes RGS5, MYH11, and ACTA2, suggesting the significant specificity of myCAFs within the TME, whereas other CAF phenotypes were more challenging to distinguish and define (**Figure 5D**). Prior to immunotherapy, the abundance of NDUFA4L2^+^ CAF subpopulation was relatively high but significantly decreased after treatment (**Figure 5E**). However, this cell subpopulation still retained a proportion within the CAF population of post-treatment patients (**Figure 5E**). Enrichment analysis revealed that NDUFA4L2^+^ CAF subpopulation was enriched in pathways such as oxidative phosphorylation, ECM-receptor interaction, the TGF-beta signaling pathway, and the Wnt signaling pathway, which are associated with myofibroblast characteristics (30) (**Figure 5F**). By constructing a pseudotemporal developmental trajectory of CAFs, we found that CAFs exhibited four differentiation paths; interestingly, NDUFA4L2^+^ CAF subpopulation was positioned at the endpoints of these differentiation pathways (**Figures 5G, H**). Additionally, GRN analysis further revealed the factors with the highest transcriptional activity among the CAF subpopulations, with TP63 being a specific regulator of NDUFA4L2^+^ CAF subpopulation (**Figure 5I**). In summary, NDUFA4L2^+^ CAF subpopulation exhibited characteristics of myofibroblasts, potentially promoting HNSCC cell migration and infiltration.

### Activation of multiple energy metabolism pathways by SPP1-positive tumor-associated macrophages

**Figure 6:**
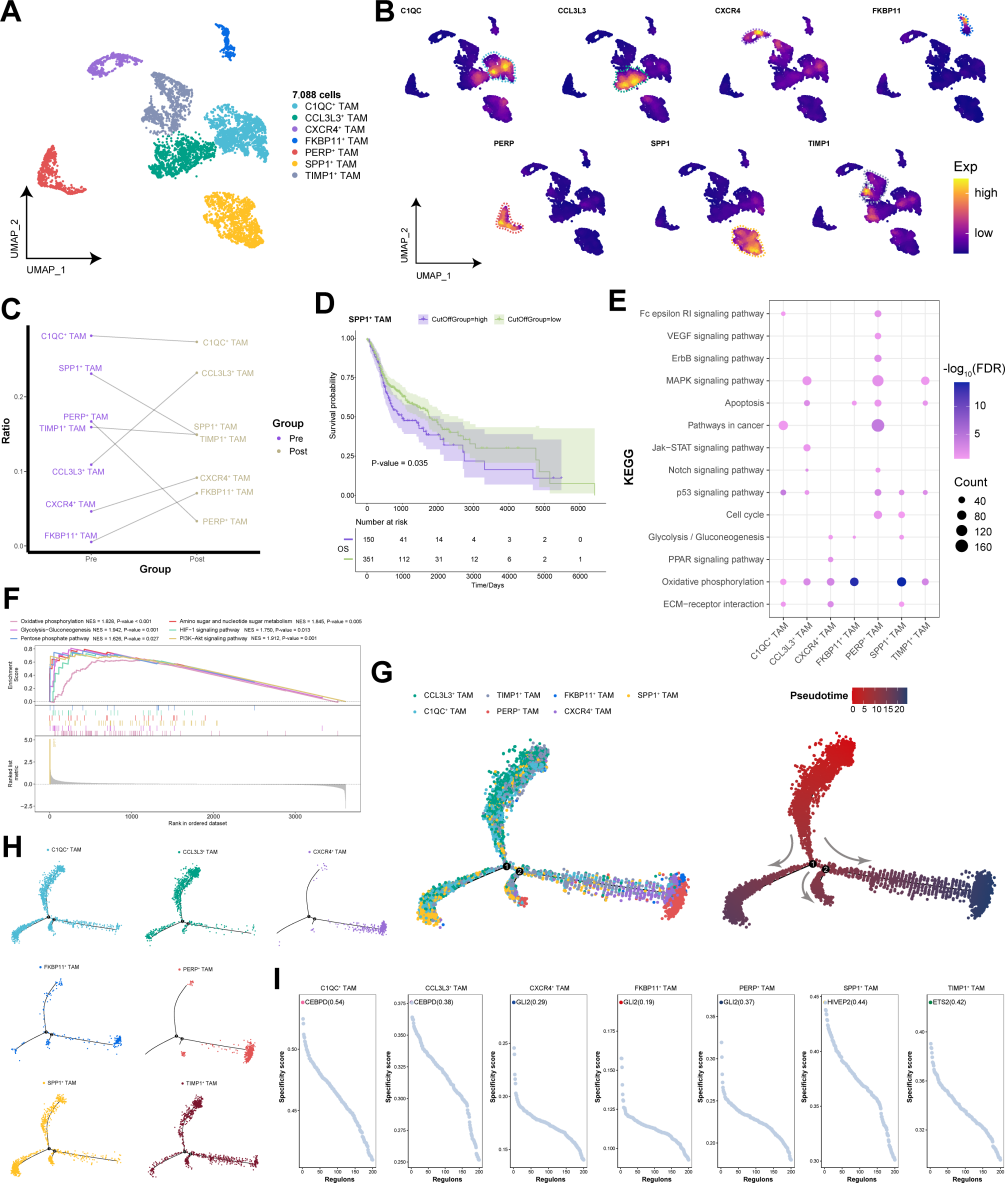
Single-cell transcriptional landscape of TAMs before and after immunotherapy. **(A)** Single-cell transcriptional landscape of TAM subpopulations. **(B)** Expression of specific genes in TAM subpopulations. **(C)** TAM subpopulation compositions of different groups. (Pre: Pre-treatment, Post: Post-treatment) **(D)** Prognostic efficacy of SPP1^+^ TAM subpopulation for OS. **(E)** The signaling pathways associated with TAM subpopulations. **(F)** Signaling pathways significantly activated by the SPP1^+^ TAM subpopulation. **(G)** Trajectory analysis of TAM subpopulations. (Left) Pseudotime analysis mapping of TAM subpopulations. (Right) Pseudotime values displaying the differentiation trajectory of TAM subpopulations. **(H)** Pseudotime analysis mapping the various TAM subpopulations. **(I)** Specific regulators with the highest transcriptional activity in different TAM subpopulations.

Tumor-associated macrophages (TAMs) hold a dominant position within the tumor microenvironment (31). We clustered 7,088 cells into seven TAM subpopulations on the basis of their gene expression profiles and annotated each subpopulation according to genes specifically expressed within them (**Figures 6A, B**). Compared with post-immunotherapy, SPP1^+^ TAM subpopulation was relatively more abundant prior to treatment and were associated with poor prognosis in HNSCC patients (**Figures 6C, D**). Additionally, SPP1^+^ TAM subpopulation was significantly enriched in key signaling pathways related to energy metabolism, the cell cycle, and genomic stability, with pronounced activation of multiple pathways related to energy metabolism and angiogenesis (**Figures 6E, F**). Pseudotime analysis revealed three distinct differentiation trajectories among TAM subpopulations, with SPP1^+^ TAM subpopulation spanning the entire process of TAM differentiation within the tumor microenvironment, indicating their involvement in the functional transition of TAMs from an immature state to a mature state (**Figures 6G, H**). Furthermore, SPP1^+^ TAM subpopulation was regulated primarily by HIVEP2 transcription modulation (**Figure 6I**), suggesting that targeting HIVEP2 to target HIVEP2 in SPP1^+^ TAM subpopulation could help mitigate TAM metabolic activity. In summary, we identified a macrophage subpopulation, SPP1^+^ TAM subpopulation, associated with poor prognosis in HNSCC patients. It activates pathways related to energy metabolism and angiogenesis, contributing to the reprogramming of the tumor microenvironment.

### The infiltration of CD8^+^ T cells in the tumor microenvironment is associated with FOS- and S100A2-positive effector T cells

**Figure 7:**
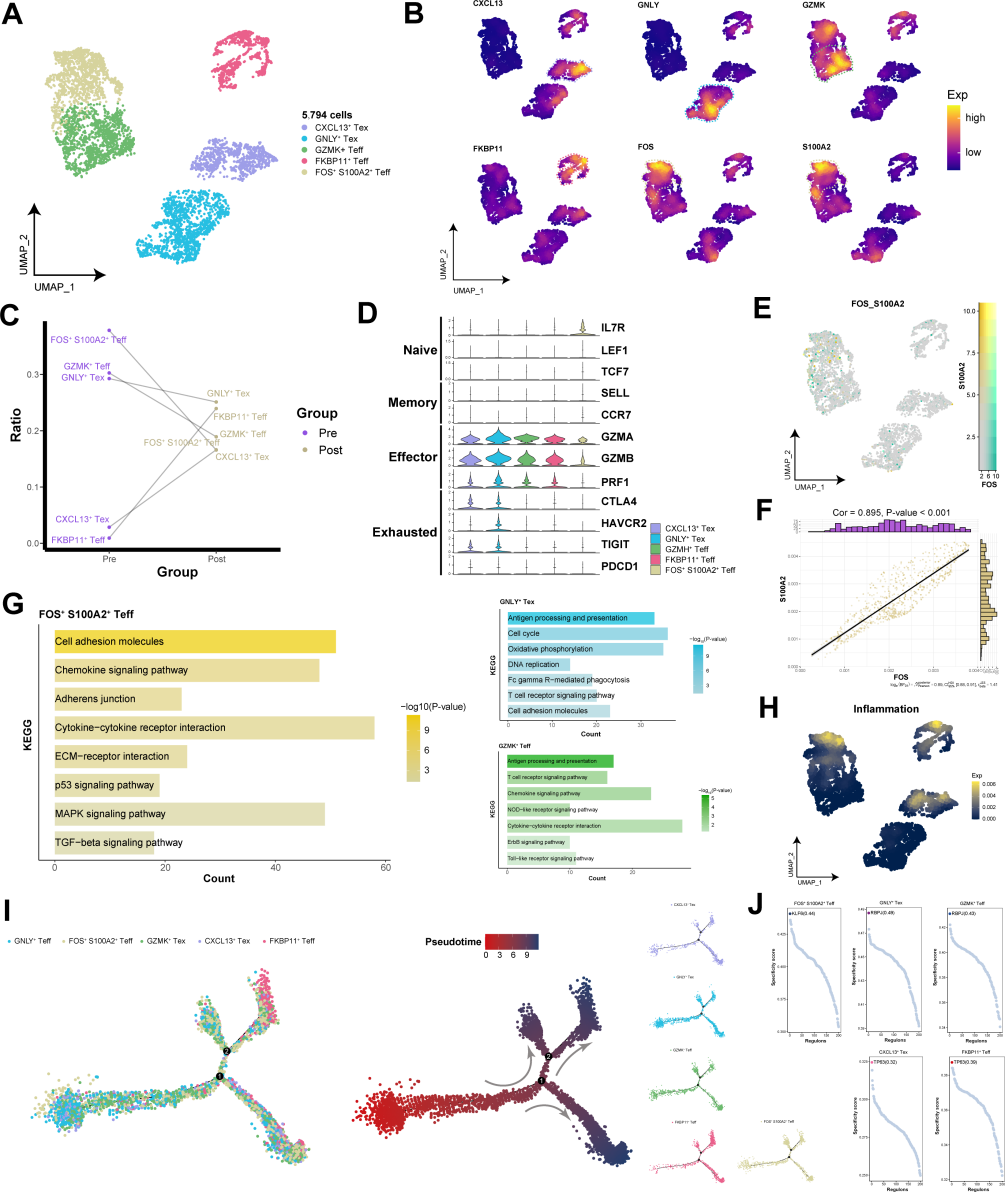
Single-cell transcriptional landscape of CD8^+^ T cells before and after immunotherapy. **(A)** Single-cell transcriptional landscape of CD8^+^ T cell subpopulations. **(B)** Expression of specific genes in CD8^+^ T cell subpopulations. **(C)** CD8^+^ T cell subpopulation compositions of the different groups. **(D)** Expression of marker genes for CD8^+^ T cell subtypes among CD8^+^ T cell subpopulations. **(E)** FOS and S100A2 coexpression in FOS^+^ S100A2^+^ Teff subpopulation. **(F)** Correlation between FOS and S100A2 in FOS^+^ S100A2^+^ Teff subpopulation. **(G)** The signaling pathways associated with CD8^+^ T cell subpopulations. **(H)** Inflammatory scores of different CD8^+^ T cell subpopulations. **(I)** Trajectory analysis of TAM subpopulations. (Left) Pseudotime analysis mapping of CD8^+^ T cell subpopulations. (Mid) Pseudotime values displaying the differentiation trajectory of CD8^+^ T cell subpopulations. (Right) Pseudotime analysis mapping the various CD8^+^ T cell subpopulations. **(J)** Specific regulators with the highest transcriptional activity in different CD8^+^ T cell subpopulations.

To explore the pivotal role of CD8^+^ T cells within the TME, we identified five subpopulations of CD8^+^ T cells and displayed their functional specificity markers through UMAP plots (**Figures 7A, B**). The changes in the CD8^+^ T cell composition across the different groups revealed that FOS^+^ S100A2^+^ Teff subpopulation was the predominant components before immunotherapy (**Figure 7C**). Interestingly, within the CD8^+^ T cell population in the TME, markers for naive and memory cells were almost not expressed, whereas a variety of effector genes were highly expressed, along with partially expressed exhausted genes (**Figure 7D**). In FOS^+^ S100A2^+^ Teff subpopulation, the coexpression of FOS and S100A2 was significantly positively correlated (Cor = 0.90) (**Figures 7E, F**). The enrichment results indicated that FOS^+^ S100A2^+^ Teff subpopulation was significantly enriched in signaling pathways related to cell migration, such as cytokine−cytokine receptor interactions, ECM−receptor interactions, and the TGF−β signaling pathway (**Figure 7G**). Moreover, FOS^+^ S100A2^+^ Teff subpopulation presented increased inflammatory factor scores (**Figure 7H**), further indicating their active state within the TME. By constructing a developmental trajectory of CD8^+^ T cells, we highlighted that all CD8^+^ T cell subpopulations were in a state of cellular maturity (**Figure 7I**). Notably, TP63 was identified as the transcription factor with the highest activity, specifically regulating CXCL13^+^ Tex and FKBP11^+^ Teff subpopulations, suggesting potential interactions between HNSCC cells and CD8^+^ T cells (**Figure 7J**). These findings further underscore the crucial role of TP63 in HNSCC. In brief, FOS^+^ S100A2^+^ Teff subpopulation may play crucial roles in regulating cellular migration, inflammatory responses, and immune reactions within the tumor microenvironment.

### The intricate cellular communication status within the tumor microenvironment

**Figure 8:**
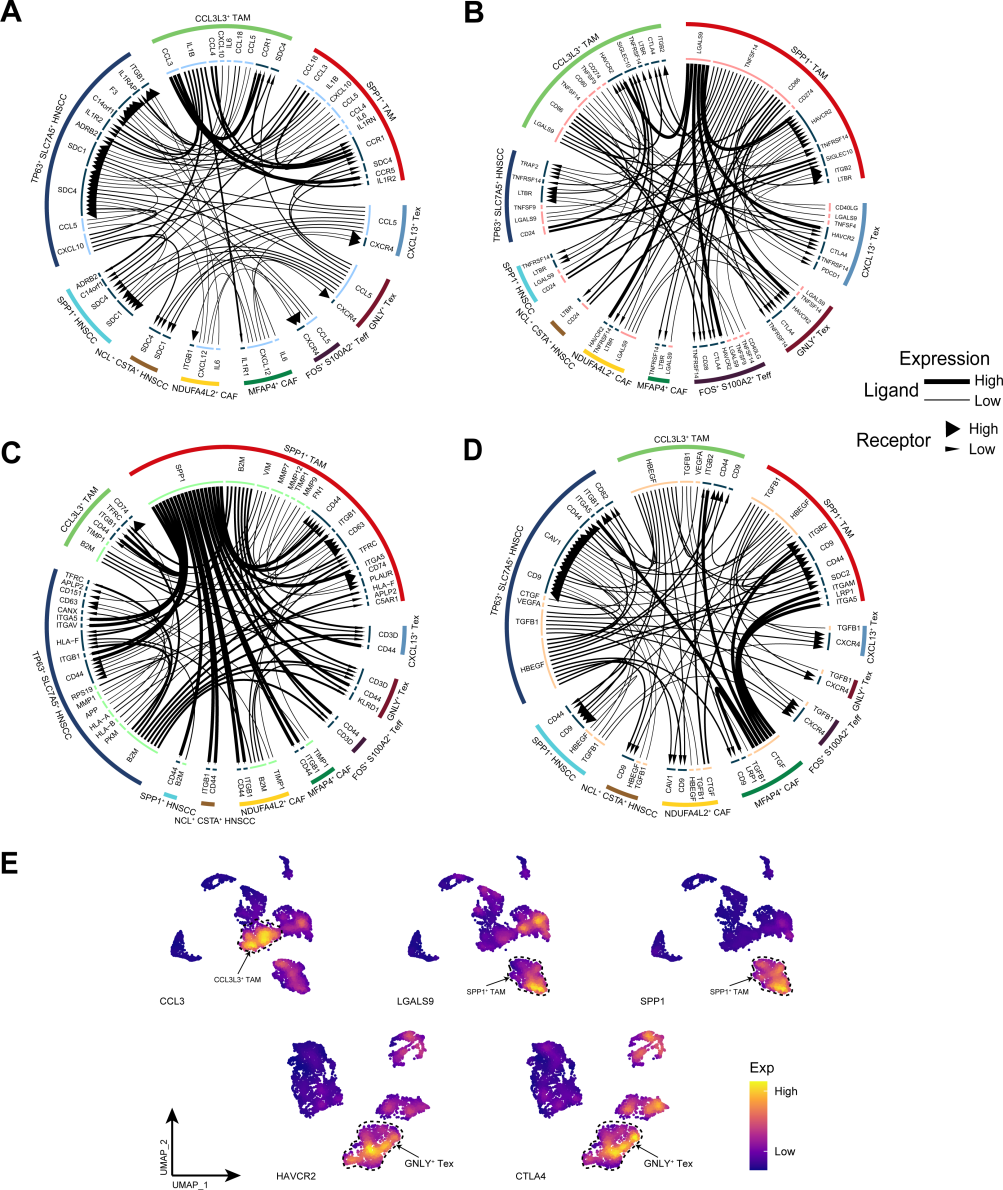
Cell communication analysis. **(A)** The cytokine receptor–ligand pairs in HNSCC. **(B)** The immune checkpoint receptor–ligand pairs in HNSCC. **(C)** The extracellular matrix receptor–ligand pairs in HNSCC. **(D)** The growth factor receptor–ligand pairs in HNSCC. **(E)** Expression of specific receptor−ligand genes.

To further investigate the communication relationships among different cellular subpopulations within the TME of HNSCC, we analyzed the interplay mechanisms of cytokines, immune checkpoints, the extracellular matrix, and growth factors. The cytokine network revealed that CCL3L3^+^ TAM subpopulation highly expressed CCL3, facilitating strong communication with both themselves and SPP1^+^ TAM subpopulation (**Figure 8A**). In the immune checkpoint network, TP63^+^ SLC7A5^+^ HNSCC subpopulation communicated with TAM subpopulations via the CD24-SIGLEC10 axis, whereas SPP1^+^ TAM subpopulation interacted with other immune cell subpopulations and CAF subpopulations through the LGALS9-HAVCR2 axis (**Figure 8B**). Within the extracellular matrix, SPP1^+^ TAM subpopulation highly expressed SPP1, which interacted with other cell subpopulations through multiple axes (**Figure 8C**). The growth factor network revealed that MFAP4^+^ CAF subpopulation interacted strongly with TAMs and TP63^+^ SLC7A5^+^ HNSCC subpopulation via CTGF (**Figure 8D**). Notably, TP63^+^ SLC7A5^+^ HNSCC subpopulation exhibited rich diversity in receptor−ligand interactions. Finally, we demonstrated the gene expression patterns of more specific receptor−ligand interaction pairs (**Figure 8E**).

## Discussion

In this study, utilizing single-cell transcriptomics data of HNSCC, we constructed a comprehensive single-cell transcriptional landscape of HNSCC. This allowed us to explore the dynamic changes and transcriptional characteristics of various cell types within the TME before and after immunotherapy and to reveal the communication crosstalk between malignant cells and immune cells. Notably, we identified a novel tumor immune escape mechanism whereby HNSCC cells may inhibit ferroptosis via TP63, thus sustaining tumor growth and differentiation. This discovery provides significant insights into how HNSCC resists current immunotherapeutic approaches.

Ferroptosis is emerging as a novel mechanism that promotes the synergistic effect of immunotherapy, radiotherapy, and chemotherapy (6). Ferroptosis is closely associated with the prognosis and progression of HNSCC and represents one of the key strategies for immunotherapy in HNSCC (32). We demonstrated that, compared with that in pre-immunotherapy conditions, the abundance of TP63^+^ SLC7A5^+^ HNSCC subpopulation increased after immunotherapy. Concurrently, TP63^+^ SLC7A5^+^ HNSCC subpopulation significantly inhibited the ferroptosis signaling pathway, which may play a crucial role in resistance to immunotherapy. TP63 acts as a lineage survival oncogene in squamous cell carcinoma (33) and is involved in the differentiation of keratinocytes and the development of squamous cells. Its encoded protein, p63, mediates epigenomic reprogramming associated with various epigenetic regulators, including chromatin remodeling complexes and epigenetic enzymes (34). SLC7A5 provides essential amino acids to cells and maintains dynamic cellular homeostasis, addressing oxidative stress by regulating ferroptosis signal transduction (35–37). In our study, there was a significant positive correlation between the coexpression of TP63 and SLC7A5 in cells, where the upregulation of TP63 protein levels led to a notable increase in SLC7A5 expression. Additionally, SLC7A5 serves as a pivotal regulatory factor in the treatment of various cancers, including multiple myeloma, breast cancer, and gastric cancer, and represents a potential therapeutic target for overcoming patient resistance (38–40). Transcriptomic data indicated that elevated SLC7A5 expression enhances the suppression of ferroptosis, particularly post-immunotherapy, with TP63^+^ SLC7A5^+^ HNSCC subpopulation demonstrating significant inhibition of the ferroptosis phenotype. Furthermore, we confirmed that in HNSCC cells overexpressing TP63, the concentrations of Fe2^+^ and ROS are significantly reduced, thereby maintaining cellular homeostasis. In summary, we propose a novel malignant cell-mediated mechanism of ferroptosis resistance: high expression of TP63 in TP63^+^ SLC7A5^+^ HNSCC subpopulation upregulates SLC7A5 expression through transcriptional regulation, inhibits ferroptosis in HNSCC cells, maintains the cellular homeostatic balance, and promotes tumor resistance to immunotherapy.

Myofibroblasts are the principal cells involved in scar formation and are ultimately responsible for excessive synthesis, deposition, and remodeling of extracellular matrix proteins in fibrosis (30). We discovered that NDUFA4L2^+^ CAF with pronounced myCAF characteristics still constitute a significant proportion after immunotherapy, suggesting the stability of this subgroup within the TME. NDUFA4L2, which is induced by hypoxia, limits the production of mitochondrial reactive oxygen species (41), aiding cells in adapting to hypoxic conditions (42). Moreover, high expression of NDUFA4L2 in hepatic fibroblasts facilitates tumor progression (43). Therefore, NDUFA4L2^+^ CAF subpopulation may not only adapt to the tumor microenvironment through NDUFA4L2 but also potentially participate in tissue remodeling and energy metabolism-related pathways to promote microenvironmental reprogramming and thus mediate tumor progression. Pancancer analysis revealed that myCAFs are more prevalent in late-stage cancers than in early-stage cancers and are potentially associated with resistance to radiotherapy and chemotherapy (29). Our study further demonstrated that myCAFs represent a terminal differentiation state of CAFs in late-stage HNSCC patients.

In our study, SPP1 was specifically expressed in SPP1^+^ TAM subpopulation, and compared with other cell subpopulations, SPP1^+^ TAM subpopulation was relatively more abundant in patients both before and after immunotherapy. SPP1 not only influences TAM polarization but is also intimately associated with the immune cell spectrum, antitumor factors, and patient prognosis (44). Reports have indicated that SPP1^+^ TAM promote angiogenesis in HNSCC and enhance tumor infiltration and metastasis through the upregulation of cytokine expression (45). Notably, we discovered that SPP1^+^ TAM subpopulation activated energy metabolism and angiogenesis-related pathways, suggesting that SPP1^+^ TAM subpopulation, by regulating energy metabolism and altering the tumor microenvironment, promote angiogenesis to supply resources for tumor growth and migration, ultimately mediating poor prognosis in HNSCC patients.

CD8^+^ T cells are terminal effectors of cancer immunity, and their effector function is indispensable in immunotherapy (46). In patients with advanced HNSCC, we observed that effector T cells constitute the main component of the CD8^+^ T cell landscape, with naive and memory T cells being almost absent, revealing potential immune regulatory dysfunctions. Despite the presence of an activated, tumor antigen-specific immune response within the HNSCC tumor microenvironment, prolonged activation can lead to T-cell exhaustion, and a reduction in memory T cells diminishes immune surveillance against the tumor, thereby facilitating immune escape. Notably, we identified an active effector T cell subpopulation, FOS^+^ S100A2^+^ Teff, which predominated among CD8^+^ T cells prior to immunotherapy. The transcription factor FOS plays a critical role in various aspects of T-cell activation programming (47, 48). S100A2 significantly participates in inflammatory cell responses and is essential for TGF-β-induced cell migration and invasion (49). Specifically, FOS^+^ S100A2^+^ Teff subpopulation was enriched in multiple cell migration-related signaling pathways, including the TGF-β signaling pathway. We speculate that FOS^+^ S100A2^+^ Teff subpopulation promote T-cell infiltration and migration toward tumor regions by increasing S100A2 levels through FOS-mediated transcriptional regulation, thereby inhibiting tumor growth and development.

Importantly, the receptors and ligands of TP63^+^ SLC7A5^+^ HNSCC subpopulation were abundant, reflecting the heterogeneity of HNSCC tumors. This richness not only renders them more sensitive to external signal stimulation but also facilitates more dynamic adaptation to the TME. Furthermore, TP63^+^ SLC7A5^+^ HNSCC subpopulation may resist phagocytosis by macrophages through the CD24-SIGLEC10 axis (50), promoting tumor escape. Previous research has indicated that CCL3 recruits immune cells to tumors and may reduce metastasis through tumor−macrophage interactions (51). We found that CCL3L3^+^ TAM subpopulation recruit additional TAM via CCL3 secretion, whereas SPP1^+^ TAM subpopulation exacerbate immune suppression in the TME by highly expressing LGALS9, which binds to the immune checkpoint molecule HAVCR2 on T cells and CAFs (52). This finding corroborates the potential of LGALS9-HAVCR2 as a target for HNSCC immunotherapy. Reports have indicated that the SPP1−CD44 axis is highly active in the communication of macrophages with other cells in colorectal cancer, where it mediates immune suppression (53). Our observations of a similar phenomenon in the extracellular matrix network of the TME further substantiate the critical role of SPP1^+^ TAM subpopulation in HNSCC. Additionally, our study identified TP63 as the most specific regulatory transcription factor for CXCL13^+^ Tex, FKBP11^+^ Teff, and NDUF4L2^+^ CAF subpopulations. These findings suggest that TP63^+^ SLC7A5^+^ HNSCC subpopulation may also regulate the function and growth of T cells and fibroblasts through the secretion of TP63, potentially via exosomes. This warrants further exploration in subsequent studies.

We utilized bioinformatics approaches based on single-cell and bulk transcriptomics data to reveal the heterogeneity of the TME in HNSCC before and after immunotherapy, complemented by preliminary experimental validation of our hypotheses. However, our results have several limitations. First, the sample size for our single-cell study cohort was small, necessitating further validation across a larger clinical sample set. Although the sample size in our study is limited, the large number of cells captured per sample provides sufficient data to uncover significant biological insights. Second, the observational nature of our current study limits the establishment of causative relationships. The molecular mechanisms underlying the TP63-mediated transcriptional regulation of SLC7A5 and the subsequent mediation of ferroptosis by SLC7A5 require further confirmation through functional experiments. We plan to incorporate functional experiments in future research to validate the key findings related to treatment response mechanisms.

## Summary

In summary, we revealed changes in the cellular ecology of the TME in HNSCC before and after immunotherapy. We discovered a novel self-protection mechanism by malignant cells: TP63-mediated transcriptional regulation of SLC7A5 expression inhibits ferroptosis in malignant cells, thereby conferring resistance to immunotherapy. This contributes to a better understanding of the mechanisms underlying the response of HNSCC to immunotherapy.

## Data Availability

The original contributions presented in the study are included in the article/**Supplementary Material**. Further inquiries can be directed to the corresponding authors.

## Supplementary material

Supplementary Figure 1 Gene expression, predicted peaks, gene set scoring, pseudotemporal gene dynamics, and regulators of highest transcriptional activity in HNSCC cell subpopulations. **(A)** Expression of specific genes in CD8^+^ T cell subpopulations. **(B)** Single-cell transcriptional landscape of HNSCC cell subpopulations in different groups. **(C)** Prediction of peaks for TP63-protein and SLC7A5-DNA binding. **(D)** Ferroptosis promotion score of HNSCC cell subpopulations. **(E)** Dynamic expression distributions of TP63 and SLC7A5 during the development of HNSCC cell subpopulations. **(F)** Specific regulators with the highest transcriptional activity in different HNSCC cell subpopulations.
