# Supplementary figures and images for: TP63 transcriptionally regulates SLC7A5 to suppress ferroptosis in head and neck squamous cell carcinoma
Source: Front Immunol. 2024 Aug 21;15:1445472. doi: 10.3389/fimmu.2024.1445472 (PMC11371717; doi:10.3389/fimmu.2024.1445472)

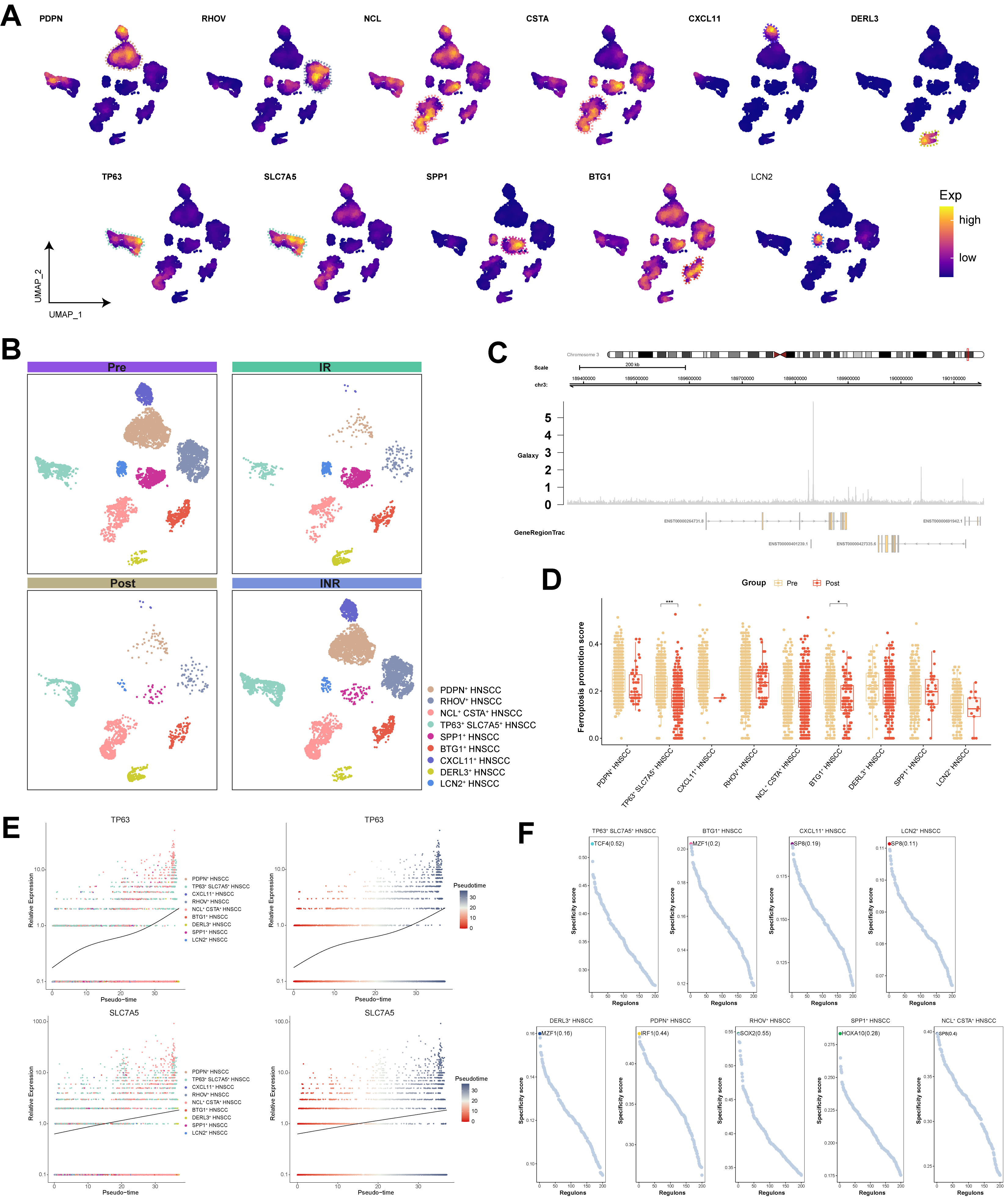

Supplement: Supplementary Figure 1 — Gene expression, predicted peaks, gene set scoring, pseudotemporal gene dynamics, and regulators of highest transcriptional activity in HNSCC cell subpopulations. (A) Expression of specific genes in CD8+ T cell subpopulations. (B) Single-cell transcriptional landscape of HNSCC cell subpopulations in different groups. (C) Prediction of peaks for TP63-protein and SLC7A5-DNA binding. (D) Ferroptosis promotion score of HNSCC cell subpopulations. (E) Dynamic expression distributions of TP63 and SLC7A5 during the development of HNSCC cell subpopulations. (F) Specific regulators with the highest transcriptional activity in different HNSCC cell subpopulations. [file Image1.tif]
